# Influence and Mechanism of Coal Gangue Sand on the Properties and Microstructure of Shotcrete Mortar

**DOI:** 10.3390/ma18091940

**Published:** 2025-04-24

**Authors:** Yong Cui

**Affiliations:** 1 CCTEG Coal Mining Research Institute, Beijing 100013, China; cuiy1224@163.com; 2 National Key Laboratory of Disaster Prevention and Ecological Protection in Open Pit Coal Mines, Beijing 100013, China

**Keywords:** coal gangue sand, shotcrete mortar, mortar setting time, water absorption, microscopic interface

## Abstract

Coal gangue, a fine aggregate for the preparation of shotcrete mortar, is a cost-effective approach for the resource utilization of coal gangue. This study employed a mortar setting time tester, electronic universal testing machine, water absorption tester, nitrogen adsorption–desorption instrument (BET), X-ray diffraction (XRD), scanning electron microscopy (SEM), and life cycle assessment (LCA) to investigate the effects and mechanisms of replacing natural sand with coal gangue sand (0–100%) under water-to-binder ratios of 0.4 and 0.55 on the macroscopic properties, microstructure, and environmental impact of shotcrete mortar. The results showed that the porous nature of coal gangue sand increased the porosity of shotcrete mortar and reduced its compressive strength. However, its water absorption effectively decreased the effective water-to-binder ratio, significantly shortening the initial setting time. At a water-to-binder ratio of 0.55, as the replacement ratio of coal gangue sand increased from 0% to 100%, the porosity of shotcrete mortar increased by approximately 30%, the compressive strength decreased by about 40%, and the initial setting time was shortened by 57%. When the water-to-binder ratio was reduced to 0.4 and the replacement ratio of coal gangue sand was 50%, the shotcrete mortar met the application requirements of M20 shotcrete mortar, with an initial setting time of less than 12 min and a compressive strength of over 23 MPa after 28 days of water curing. Microstructural analysis revealed that the absorbed water in coal gangue sand played an internal curing role during cement hardening, improving the compactness of the interfacial transition zone. Environmental assessment results indicated that, under the same strength conditions, the life cycle environmental impact of coal gangue sand shotcrete mortar was approximately 70% lower than that of natural sand shotcrete mortar. This study provides a theoretical basis for the efficient resource utilization of coal gangue and the preparation of low-carbon shotcrete mortar.

## 1. Introduction

Coal is an important component of the global energy structure, and coal gangue, generated during its mining and washing, has become a widely distributed industrial solid waste [1,2,3]. It is reported that the annual emission of coal gangue in China amounts to 200 million tons, with cumulative storage exceeding 6 billion tons [4,5]. This large-scale accumulation not only occupies land resources but also poses a severe threat to the ecological environment due to the release of harmful gases such as SOx and NOx and the leaching of heavy metal ions [6,7]. It is imperative to increase the utilization rate of coal gangue and reduce its stockpile.

The main mineral components of coal gangue include kaolinite, illite, quartz, mullite, montmorillonite, etc. [3]. Although calcination treatment can decompose some aluminosilicate minerals, forming amorphous phases with pozzolanic activity, the differences in regional geological origins lead to significant fluctuations in the active components of coal gangue from different mining areas [8]. Particularly, when the content of inert minerals like quartz and mullite is high, its reactivity as a supplementary cementitious material often fails to meet the requirements of Chinese standard GB/T 2847-2022, making it difficult to consume large amounts of coal gangue. In contrast, the chemical composition of coal gangue is relatively stable, with the typical components being SiO_2_ and Al_2_O_3_, which provide a theoretical basis for its use as concrete aggregate [3]. However, the defects of coal gangue aggregate are also apparent. Coal gangue contains a small amount of organic matter and fragile components such as expansive clay, which affect the hydration of cement and reduce the strength of concrete [9,10,11]. Furthermore, mechanical mining and crushing cause microcracks within coal gangue particles, lowering their particle strength. Studies show that concrete prepared with coal gangue exhibits reduced elastic modulus and increased shrinkage, with overall performance generally inferior to ordinary C30 concrete [3,12].

The preparation of manufactured sand from coal gangue represents a novel approach to coal gangue resource utilization, possessing the potential to replace natural river sand due to its similar chemical composition [4]. This method not only mitigates the issue of over-exploitation of natural river sand resources but also alleviates the adverse effects of internal cracks in coal gangue on concrete performance through particle size control. Yu et al. [4] indicated that using a 25% coal gangue sand replacement rate can enhance the mechanical properties of mortar, but complete replacement leads to a significant reduction in mortar strength. Zhang et al. [13] suggested that the irregular appearance of coal gangue sand could improve the strength and stiffness of concrete. However, such angular particles are more prone to stress concentration effects, accelerating the formation and propagation of cracks [14]. Considering that their study subjects were reinforced concrete, this negative effect might be more pronounced in non-reinforced mortars. Qiu et al. [15] found that when the replacement rate of coal gangue sand for natural sand is within 40%, concrete exhibits good freeze–thaw resistance and optimized pore structure, which is related to the secondary hydration of coal gangue sand. However, Hao et al. [16] showed that replacing natural sand with coal gangue sand significantly reduces concrete fluidity. In summary, while coal gangue sand has the potential to replace natural sand, its application scope needs to be expanded to fully utilize its advantages.

Shotcrete technology, a mature engineering technique, is widely applied in slope reinforcement, municipal infrastructure, and tunnel construction [17,18]. Applying coal gangue sand in shotcrete mortar may offer new avenues for enhancing coal gangue resource utilization. Chen et al. [19] studied the effects of different coal gangue sand contents on the compressive strength and durability of shotcrete, finding that the compressive strength of shotcrete depends not only on the content of coal gangue but also on the water–cement ratio. Zhang et al. [20] modified coal gangue sand using nano-silica, improving the interface bond strength and mechanical properties of shotcrete. They also discovered that incorporating 71% coal gangue sand reduced the total energy consumption and CO_2_ emissions of shotcrete by 24–32% and 12–24%, respectively. For shotcrete mortar, in addition to fluidity and mechanical properties, attention must be paid to the setting time of the mortar. This is because, compared to the setting time of cement paste, the setting time of mortar better reflects the setting characteristics of the mortar itself and is closely related to the bonding condition with the stressed surface and the rebound rate after spraying. Moreover, the porous nature of coal gangue sand significantly influences the macro- and micro-interface of shotcrete mortar during the mixing and curing stages due to its water absorption properties, but research on the mechanisms of these effects remains insufficient.

To address the above issues, this study systematically investigated the effects of coal gangue sand content (0–100%) on the macro- and micro-properties of shotcrete mortar under different water–cement ratios using a mortar setting time tester, universal mechanical testing machine, water absorption tester, nitrogen adsorption–desorption isotherm analyzer (BET), X-ray diffractometer (XRD), and scanning electron microscope (SEM). In addition, this study assessed the environmental performance of coal gangue sand shotcrete concrete using the life cycle assessment (LCA) method.

## 2. Experimental Program

### 2.1. Materials

**Figure 1:**
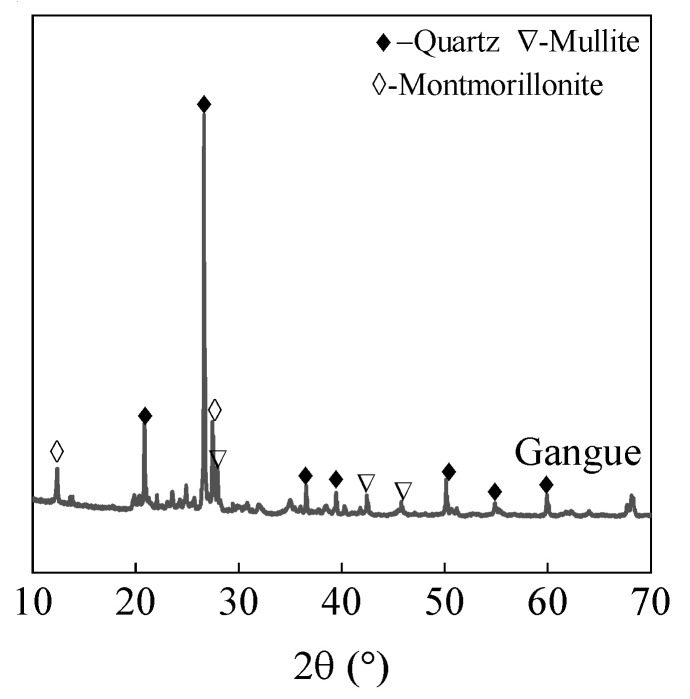
Mineral composition of coal gangue.

**Figure 2:**
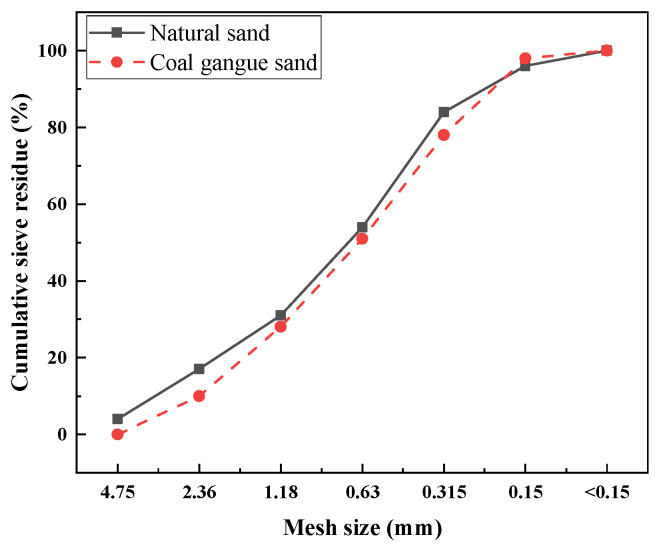
Particle size distribution of coal gangue sand and natural sand.

**Figure 3:**
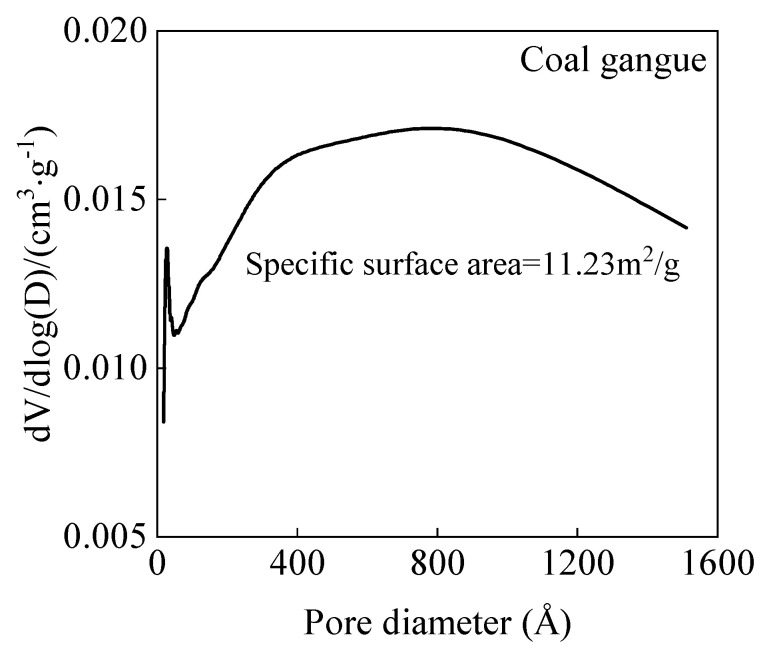
The BET results of coal gangue sand.

The cement (Conch Co., Ltd., Xuancheng, China) used was P.O. 42.5 ordinary Portland cement. The natural sand is provided by Jiuqi Building Materials Company, Zhucheng, China. The coal gangue was sourced from the Laogongyingzi Coal Mine of Pingzhuang Coal Industry (Group) Co., Ltd., Chifeng, China. The chemical and mineral compositions of natural sand and coal gangue sand are shown in Table 1 and Figure 1, respectively. The main minerals in coal gangue sand were inert minerals such as quartz, montmorillonite, and mullite, indicating that coal gangue sand possesses a certain level of stability. Figure 2 presents the particle size distribution of natural sand and coal gangue sand, and the fineness modulus of coal gangue sand and natural sand is 2.65 and 2.82, respectively. According to the Chinese standard GB/T 14684-2022, both sands are classified as medium sand, with natural sand being coarser than coal gangue sand. The bulk density of coal gangue sand is 1350 kg/m^3^, with a water absorption rate of 6.5%, while the bulk density of natural river sand is 1500 kg/m^3^, with a water absorption rate of 1.1%. Figure 3 shows the BET test results of coal gangue sand, which has a specific surface area of 11.23 m^2^/g. This result is consistent with previous studies indicating the porous nature of coal gangue sand. The accelerator used was a J85-type accelerator (Xinda New Materials Co., Ltd. Huaibei, China) (main chemical components are Al_2_O_3_ and SiO_2_), a polycarboxylate superplasticizer was used as the water reducer (Shanxi Feike New Materials Technology Co., Ltd. Yuncheng, China), and the water used is tap water.

### 2.2. Sample Preparation

The mix proportions of the shotcrete mortar test are shown in Table 2. In the shotcrete mortar system with water–cement ratios of 0.55 and 0.4, the effect of coal gangue sand content on the setting time, compressive strength, water absorption, and microstructure of shotcrete mortar was investigated by adjusting the mass ratio of coal gangue sand replacing natural sand (replacement ratio ranging from 0% to 100%).

According to the mix proportions in Table 2, cement, coal gangue sand, and natural sand were first mixed for 2 min, followed by the addition of the corresponding amount of water and mixing for another 5 min to form fresh mortar. The fresh mortar was then poured into 40 × 40 × 160 mm^3^ triple molds, vibrated on a vibration table for 30–60 s, leveled off, and covered with plastic wrap. The molds were cured for 1 day before demolding, after which the specimens were placed in water at 20 ± 2 °C for curing for up to 14 and 28 d.

### 2.3. Test Method

#### 2.3.1. Setting Time

**Figure 4:**
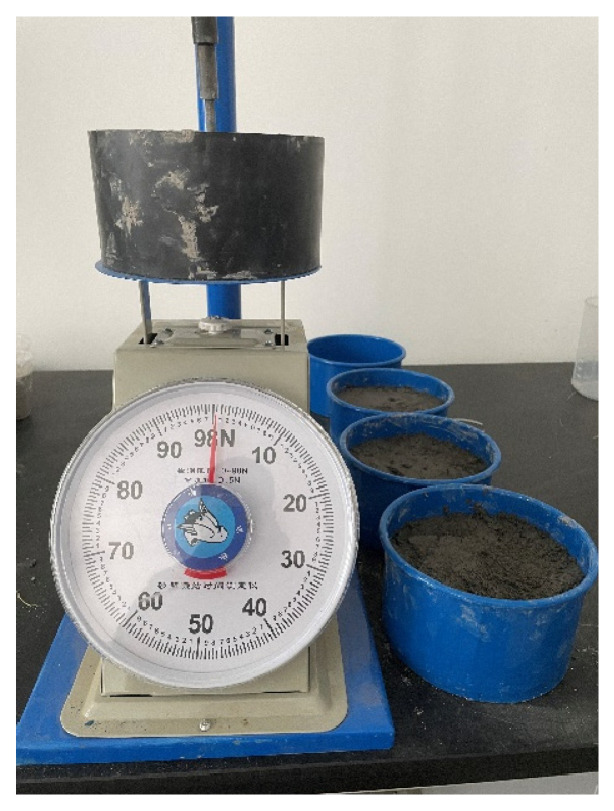
Setting time tester of mortar.

According to Chinese standard JGJ/T 70-2009, the setting time of mortar was tested as follows, as shown in Figure 4. The prepared mortar mixture was placed into a mortar container, which was then positioned on the pressure gauge’s circular disk. The adjustment nut was rotated to ensure that the penetration depth of the needle into the mortar was 25 mm, and the nut was then fixed in place. The pressure rod was manually pressed vertically downward, and within 10 s, the test needle was penetrated 25 mm into the mortar. The reading shown on the pressure gauge at this point was recorded as the first measurement. This process was repeated every 5 min until the resistance value reached 0.7 MPa. The penetration resistance value was calculated using the following formula:

(1) $$f_{p}=\frac{N_{p}}{A_{p}}$$

where $f_{p}$ is the penetration resistance value (MPa), $N_{p}$ is the static pressure (N) when the penetration depth is 25 mm, and $A_{p}$ is the cross-sectional area of the penetration needle (30 mm^2^).

#### 2.3.2. Compressive Strength Test

According to Chinese standard GB/T 17671-2021, the compressive strength of shotcrete mortar was tested as follows. The compressive strength test was conducted on three specimens. Each specimen was subjected to a compressive load until failure, and the corresponding compressive strength value was recorded for each specimen. The compressive strength of the shotcrete mortar was calculated as the average value of the compressive strength measurements of the three specimens.

#### 2.3.3. Water Absorption Test

The specimen was fully immersed in water, ensuring that all surfaces were covered by water. After 1 day of curing, the initial mass of the specimen was measured and recorded. After curing for 14 days and 28 days, the specimen was removed, and the surface water was wiped off before measuring its mass (*G_t_*). The specimens were then dried at 40 °C for 48 h to obtain the mass (*G*_0_). The water absorption rate was calculated using the following formula:

(2) $$\varphi_{t}=\frac{G_{t}-G_{0}}{G_{0}}$$

where $\varphi_{t}$ represents the water absorption rate (%), $G_{0}$ is the initial mass of the specimen after drying (g), and $G_{t}$ is the mass of the specimen at specific time points (g).

#### 2.3.4. Microscopic Characterization

Samples (5 mm × 5 mm × 5 mm) were taken from different specimens, soaked in anhydrous alcohol for 48 h to terminate the reactions, and then placed in a 40 °C oven for 48 h. Before X-ray diffraction analysis (XRD), the sample particles had to be ground to a powder. For scanning electron microscopy (SEM) and nitrogen adsorption–desorption (BET) tests, the dried small sample blocks were used directly.

## 3. Result and Discussion

### 3.1. Surface Condition

**Figure 5:**
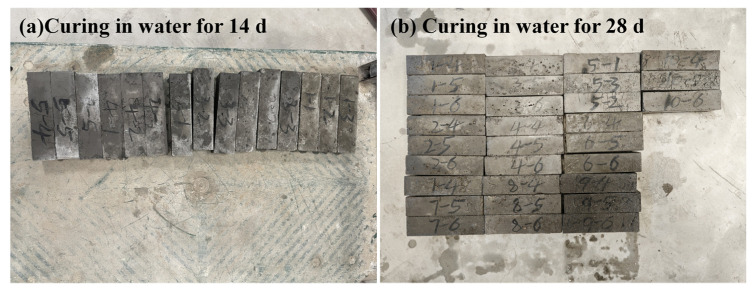
Surface of coal gangue sand shotcrete mortar after soaking in water at different ages.

Figure 5 shows the surface images of coal gangue sand shotcrete after being soaked in water for different curing periods. It can be observed that shotcrete with varying coal gangue sand content did not exhibit corner chipping or cracking after 14 and 28 days of water immersion. Even when fine aggregates were entirely replaced with coal gangue sand, no corner chipping or cracking occurred. Shotcrete is primarily used for reinforcing surrounding rock in underground spaces and often interacts with groundwater fractures. Whether the specimen deforms after water immersion determines whether coal gangue can be used in shotcrete. After water immersion, the crystal expansion forces of these components change significantly, and the original crystal structure is destroyed, which macroscopically manifests as expansion deformation and disintegration of coal gangue. This study found that the surface of shotcrete prepared with coal gangue sand as fine aggregate did not exhibit bending or cracking, indicating the preliminary feasibility of using coal gangue sand in shotcrete preparation.

### 3.2. Setting Time

**Figure 6:**
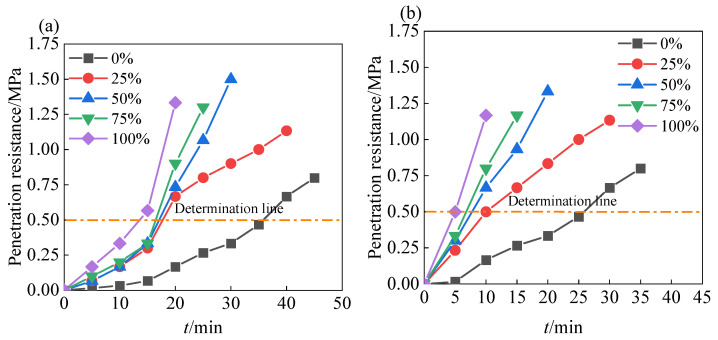
Effect of different coal gangue substitution rates on the setting time of shotcrete mortar under different water–cement ratios: (**a**) water–cement ratio of 0.55; (**b**) water–cement ratio 0.4.

Figure 6 illustrates the effect of coal gangue sand content on the setting time of shotcrete under different water–cement ratios. The results show that both the water–cement ratio and the amount of coal gangue sand significantly affect the setting time of shotcrete. When the water–cement ratio decreased from 0.55 to 0.4, the setting time of shotcrete containing only natural sand was reduced to about 25 min. This is because a lower water–cement ratio reduces the amount of free water in the mortar system, decreases the spacing between cement particles, and allows hydration products to fill the pores more quickly, forming a denser structure [18,21].

In the system with a water–cement ratio of 0.55 (Figure 6a), as the coal gangue sand content increased from 0% to 100%, the setting time of shotcrete significantly decreased from 40 min to about 15 min. This indicates that the addition of coal gangue sand significantly accelerates the setting process. This is mainly due to the abundant fine particles and mineral components such as montmorillonite in coal gangue sand, which have a strong water adsorption capacity, significantly reducing the effective water–cement ratio in the mortar system and thereby accelerating the cement hydration reaction [18,22,23]. Additionally, the porous structure of coal gangue sand may provide more hydration reaction interfaces for cement particles, further enhancing the reaction rate of cement [23].

In the system with a water–cement ratio of 0.4 (Figure 6b), as the coal gangue sand content increased from 0% to 100%, the setting time of shotcrete further decreased from 25 min to about 5 min. Under low water–cement ratio conditions, the adsorption effect and porous characteristics of coal gangue sand have a more significant impact on promoting the cement hydration reaction. In low water–cement ratio systems, the amount of free water is already low, and the introduction of coal gangue sand further reduces the effective water–cement ratio, making the hydration reaction faster and more concentrated [24]. Meanwhile, the surface effect of the porous structure is fully utilized under low water–cement ratio conditions, providing more reaction interfaces for cement particles and significantly accelerating the setting process [23]. This characteristic of coal gangue sand in accelerating mortar setting helps reduce the amount of accelerating agent used, thereby improving the economic and environmental friendliness of shotcrete.

### 3.3. Compressive Strength

**Figure 7:**
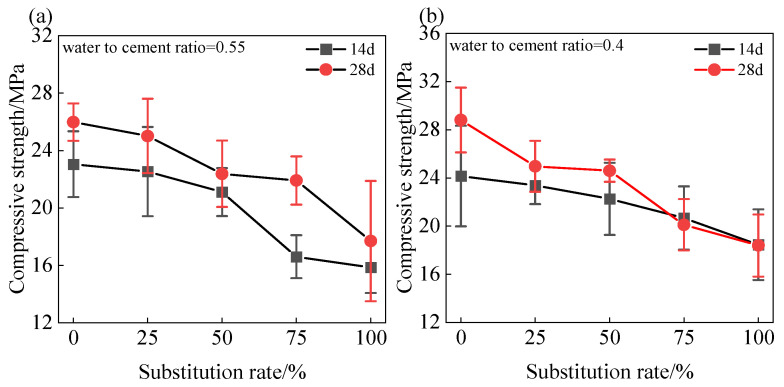
Effect of different coal gangue substitution rates on the compressive strength of shotcrete mortar under different water cement ratios: (**a**) water–cement ratio of 0.55; (**b**) water–cement ratio 0.4.

Figure 7 shows the effect of coal gangue sand content on the compressive strength of shotcrete under different water–cement ratios. The results indicate that both an increase in the water–cement ratio and the coal gangue sand content lead to a decrease in the compressive strength of shotcrete, with this effect exhibiting certain regularities across different curing periods and water–cement ratios.

When the water–cement ratio was 0.55, as the coal gangue sand content increased from 0% to 100%, the 14-day compressive strength of shotcrete decreased from 23.0 MPa to 15.9 MPa, and the 28-day compressive strength decreased from 26 MPa to 17.7 MPa. For a water–cement ratio of 0.4, as the coal gangue sand content increased from 0% to 100%, the 14-day compressive strength of shotcrete decreased from 24.2 MPa to 18.5 MPa, and the 28-day compressive strength decreased from 28.8 MPa to 18.4 MPa.

Reducing the water–cement ratio is generally considered an effective way to improve the mechanical properties of mortar [25]. This is because a lower water–cement ratio reduces the porosity of the mortar, enhances the density of the cement paste, and thereby increases the compressive strength. However, when coal gangue sand was used to replace natural sand, the overall mechanical properties declined, despite the reduction in the effective water–cement ratio of the mortar. The reasons for this phenomenon may be as follows. (1) The particle size distribution of coal gangue sand is suboptimal. Relative to natural sand, coal gangue sand exhibits inferior particle grading, deviating from the dense packing theory’s requirement for efficient particle interlock and void minimization [26]. This increases the internal porosity of the mortar, weakens its load-bearing capacity, and provides pathways for crack propagation, significantly affecting the compressive strength of the mortar. (2) The particles of coal gangue sand are irregular [12]. Although the rough particle surface can adsorb more free water, which aids in the early setting of the mortar, it may also lead to uneven distribution of cement paste on the particle surface, forming a poor-quality interfacial transition zone. The irregular morphology of coal gangue sand induces localized stress concentration in the mortar, leading to microcrack initiation and propagation, thereby increasing its susceptibility to compressive failure. (3) The particle strength of coal gangue sand is relatively low [12]. The particle strength of coal gangue sand is significantly lower than that of natural sand, especially since the strength of mineral particles such as montmorillonite and kaolinite in coal gangue sand is far inferior to that of quartz and feldspar in natural sand [27]. Under compressive loads, coal gangue sand particles are often the first to break or deform, thereby reducing the overall strength of the mortar.

Furthermore, in terms of strength development, mortars with high coal gangue sand replacement rates exhibited more significant strength growth under high water–cement ratio conditions. This indicates that under low water–cement ratio conditions, a high coal gangue sand content may result in insufficient water for hydration reactions in the mortar, thereby affecting the hydration and strength development of the cement paste.

### 3.4. Water Absorption Rate

**Figure 8:**
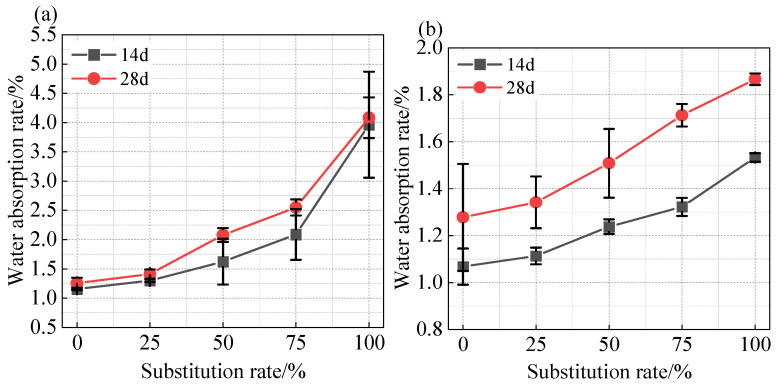
Effect of different coal gangue substitution rates on the water absorption of shotcrete mortar under different water cement ratios: (**a**) water–cement ratio of 0.55; (**b**) water–cement ratio 0.4.

The water absorption rate of shotcrete is an important indicator for evaluating its pore structure and density. A higher water absorption rate generally indicates greater internal porosity, stronger connectivity, and lower density of the mortar [28,29]. Figure 8 shows the influence of different coal gangue sand contents on the water absorption rate of shotcrete under different water–cement ratios.

In Figure 8a, it can be seen that when the water–cement ratio was 0.55, as the coal gangue sand content increased, the 14-day water absorption rate of shotcrete significantly rose from 1.2% to about 4%. As the curing age increased, the water absorption rate of the shotcrete increased slightly, but the growth rate was limited. This was mainly because, under a water–cement ratio of 0.55, the mortar had high porosity and strong connectivity between pores. During the early hydration process, the pores, cracks, and coal gangue sand particles in the mortar quickly approached saturation, thereby limiting the growth rate of water absorption in the later stages.

When the water–cement ratio decreased to 0.4, the water absorption rate of shotcrete still increased with the increase in coal gangue sand content. However, compared to the system with a water–cement ratio of 0.55, the overall water absorption rate of shotcrete with a water–cement ratio of 0.4 decreased by about 50%. This is because a reduction in the water–cement ratio significantly decreases the free water content in the mortar, thereby refining the pore structure of the mortar. Studies have shown that a lower water–cement ratio can effectively reduce the connectivity between pores and decrease the rate of water transport within the pores [25,28]. Therefore, the water absorption rate of shotcrete in low water–cement ratio systems is significantly lower than that in high water–cement ratio systems. Meanwhile, under low water–cement ratio conditions, due to the lack of free water, a large number of cement particles undergo hydration reactions on the surface of coal gangue sand particles, forming a hydration product layer that envelops the coal gangue sand particles. This enveloping layer can effectively block direct contact between coal gangue sand and water, reducing the water absorption of coal gangue sand and thereby lowering the overall water absorption of the mortar. As the curing time extended, free water gradually entered the pores and cracks in the mortar and penetrated the coal gangue sand, resulting in a larger increase in its water absorption rate.

### 3.5. Microscopic Characterization

#### 3.5.1. BET

**Figure 9:**
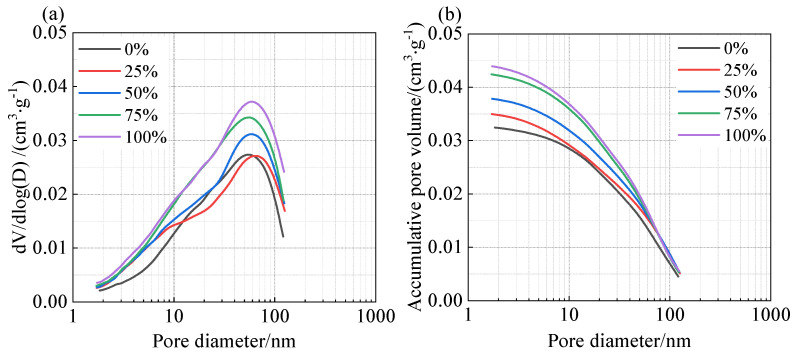
Effect of coal gangue substitution rates on the pore structure of shotcrete mortar under a water-to-cement ratio of 0.4, (**a**) differential pore distribution; (**b**) cumulative pore distribution.

N_2_ adsorption–desorption isotherm testing is a commonly used microscopic characterization method to analyze the pore size distribution and pore structure characteristics of materials through adsorption isotherms. This method not only reveals the microscopic characteristics of materials but also avoids damage to the sample structure caused by the low strength of coal gangue. Figure 9 shows the effect of different coal gangue sand content on the pore structure of shotcrete under a water–cement ratio of 0.4.

In Figure 9a, it can be seen that as the coal gangue sand content increased, the peak of the pore size distribution curve of shotcrete gradually rose, the pore size distribution shifted towards larger pore sizes, and the most probable pore size of shotcrete increased from 50 nm to 60 nm. This indicates that the incorporation of coal gangue sand leads to an increase in the proportion of mesopores in the mortar, and the pore structure becomes coarser.

As the coal gangue sand content increased (as shown in Figure 9b), the cumulative pore volume of shotcrete showed an overall increasing trend, indicating that the incorporation of coal gangue sand significantly increased the total porosity of the mortar. These microscopic structural characteristics are consistent with the increase in porosity observed in the water absorption rate tests, indicating that the porous nature and rough surface of coal gangue sand particles are the main reasons for the increase in mortar porosity.

#### 3.5.2. XRD

**Figure 10:**
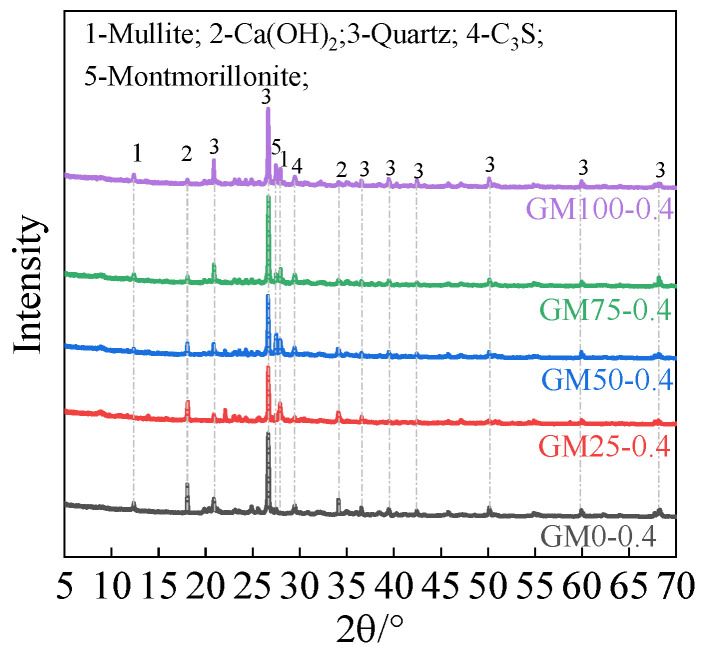
Effect of coal gangue substitution rates on the mineral composition of shotcrete mortar under a water–cement ratio of 0.4.

Figure 10 shows the XRD patterns of mortar after 28 days of curing under a water–cement ratio of 0.4. It can be observed that the main crystalline phases in the mortar include calcium hydroxide, quartz, tricalcium silicate, and montmorillonite [27]. Among these, tricalcium silicate is the primary mineral component in cement, producing calcium hydroxide and C-S-H gel during the hydration process [23], while quartz and montmorillonite are inherent mineral components in coal gangue sand and natural sand.

As the coal gangue sand content increased, the diffraction peak intensity of montmorillonite, a mineral unique to coal gangue sand, became stronger. Montmorillonite, a layered silicate mineral, has strong water absorption and a large specific surface area; thus, increasing the coal gangue sand content raises the overall water absorption rate of the mortar. Meanwhile, the diffraction peak intensity of the hydration product calcium hydroxide gradually decreased with the increase in coal gangue sand content. This is because coal gangue sand contains a large amount of amorphous minerals with pozzolanic activity, which can react with calcium hydroxide in secondary reactions to form additional hydration products such as C-S-H gel [23]. This pozzolanic reaction not only consumes calcium hydroxide but also further improves the microscopic structure and mechanical properties of the mortar. However, from the results of mechanical performance tests, this positive effect was insufficient to completely offset the negative impact of the porous and poor gradation of coal gangue sand on the strength of the mortar. Therefore, the content of coal gangue sand must be strictly controlled during use.

#### 3.5.3. Macro-Micro Interface Analysis

**Figure 11:**
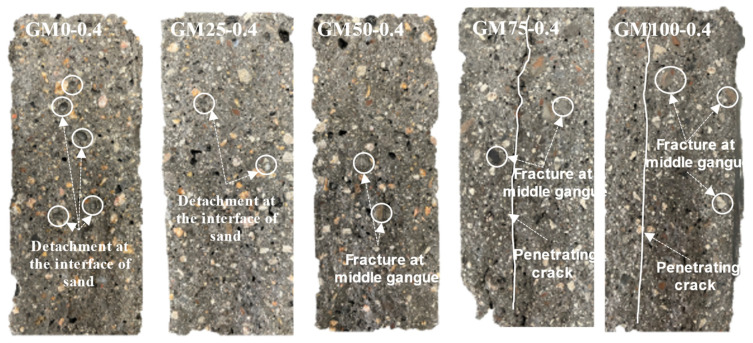
Macroscopic interface rupture of coal gangue shotcrete mortar under a water-to-cement ratio of 0.4.

Figure 11 shows the macro-interface fracture of coal gangue shotcrete mortar under a water–cement ratio of 0.4 after strength testing. It can be observed from the figure that when the shotcrete mortar contains only natural sand, numerous pits caused by the detachment of natural sand appear on the macro fracture surface. This is because the particle strength of natural sand is relatively high, and during strength testing, stress concentrates in the transition zone of the mortar interface, causing the transition zone to fail first, resulting in the detachment of natural sand particles and the formation of visible pits.

However, as the coal gangue sand content increases, the fracture mode changes significantly, with stress gradually shifting from the interface transition zone to the coal gangue sand particles themselves, leading to fractures within the particles. Particularly, when the coal gangue sand content exceeds 50%, penetrating cracks appear on the macro fracture surface of the mortar. This is because the high content of coal gangue sand particles has lower strength and is uniformly distributed within the mortar. When the mortar is subjected to pressure, the coal gangue sand particles are the first to fail due to insufficient strength. Meanwhile, the damaged particles further intensify stress concentration at the crack tips, ultimately leading to crack propagation and the formation of penetrating cracks.

**Figure 12:**
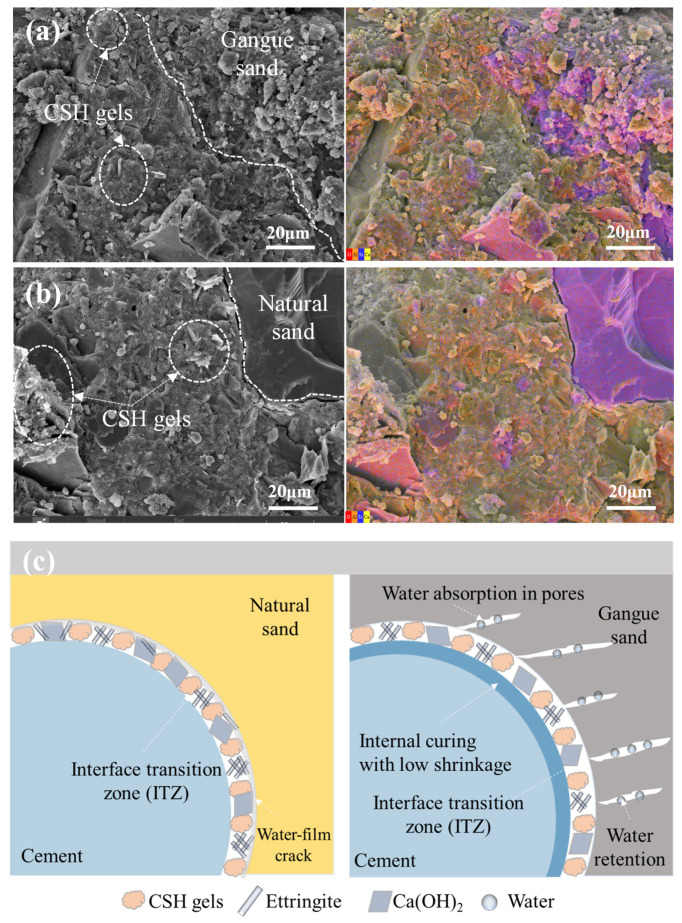
Macroscopic interface of coal gangue shotcrete mortar under a water-to-cement ratio of 0.4: (**a**) GM0-0.4, (**b**) GM100-0.4, (**c**) mechanism diagram.

Figure 12 exhibits the micro-interface of coal gangue shotcrete mortar under a water–cement ratio of 0.4. As shown in Figure 12a, when the shotcrete mortar contains only natural sand, a distinct crack appears at the junction of the interface transition zone between the natural sand and the hardened paste. The formation of such cracks is mainly due to the non-water-absorbing property of natural sand, which causes water to accumulate around its particles, creating a water film effect. The presence of the water film not only weakens the bonding performance of the interface transition zone but also leads to cracks during the later hardening process due to cement shrinkage.

In contrast, the interface transition zone between the hardened paste and coal gangue sand is more tightly connected (as shown in Figure 12b), confirming the improvement effect of coal gangue sand’s high-water absorption on the transition zone. During the cement hardening process, coal gangue sand can absorb and slowly release water, alleviating the problem of rapid internal humidity changes in the mortar to some extent. Meanwhile, the slow release of water facilitates the growth of hydration products closely adhering to the coal gangue sand particles, further reducing the width of the interface transition zone between the cement and particles.

The mechanism diagram of how coal gangue sand’s water absorption improves the interface transition zone is shown in Figure 12c.

### 3.6. Environment Assessment

Life Cycle Assessment (LCA) is a widely used method for evaluating the environmental impact (EI) of products, including Global Warming Potential (GWP), Acidification Potential (AP), Ozone Depletion Potential (ODP), Eutrophication Potential (EP), Photochemical Ozone Creation Potential (POCP), Human Toxicity Potential (HTP) of dichlorobenzene, and Abiotic Depletion Potential (ADP) [30,31]. Due to the low relevance of other indicators, this study only considers Global Warming Potential (GWP) and Acidification Potential (AP), with specific calculations shown in Equations (3)–(6). The pollutant gas emissions and potential factors during the life cycle of producing coal gangue sand shotcrete mortar are shown in Table 3 [13,32]. Based on Table 3 and Equations (3) and (4), the calculated Global Warming Potential and Acidification Potential for cement, natural sand, and coal gangue sand are shown in Table 4.

Using Table 4, Equations (5) and (6), and weight coefficients [13], the impact of different coal gangue sand contents on environmental impact coefficients and environmental strength ratio efficiency is shown in Table 5. From Table 5, it can be seen that as the coal gangue sand content increases, the environmental impact coefficient increases by about 9%, which differs from the findings of Chen et al. [19]. This is because they did not consider the gas emissions from coal gangue production and grinding during the life cycle. The difference in research results lies in the fact that the environmental impact coefficient does not account for the influence of strength. To standardize the environmental impact per unit strength, the environmental strength ratio efficiency is introduced. As the coal gangue sand content increases, the environmental strength ratio efficiency increases by about 70%. This means that at the same strength, the environmental impact of coal gangue sand shotcrete mortar is 70% lower than that of natural sand, consistent with the findings of Zhang et al. [13].

(3) $${GWP}_{j}=\sum_{i,j}{GWP}_{i,j}\times E_{i,j}$$

(4) $${AP}_{j}=\sum_{i,j}{AP}_{i,j}\times F_{i,j}$$

(5) $${WTP}_{total}=\sum_{j}{AP}_{j}\times {\varphi_{j}\times W}_{AP}+\sum_{j}{GWP}_{total}\times \varphi_{j}\times W_{GWP}$$

(6) $$k_{E-F}=\frac{{WTP}_{total}}{F_{28d}}$$

where ${WTP}_{total}$ represents the environmental impact coefficient, using social willingness to pay as an indicator; ${GWP}_{i,j}$ is the global warming potential factor of the *i* greenhouse gas in the *j* material; $E_{i,j}$ is the emission of the *i* greenhouse gas in the *j* material; ${AP}_{i,j}$ is the acidification potential factor of the *i* acidification gas in the *j* material; $F_{i,j}$ is the emission of the *i* acidification gas in the *j* material; $\varphi_{j}$ is the mass fraction of the *j* material; $W_{GWP}$ is the weighting coefficient of global warming potential factors for environmental impact; $W_{AP}$ is the weighting coefficient of acidification potential factors for environmental impact; *j* represents cement, natural sand, and coal gangue sand; $k_{M-E}$ is the environmental strength ratio efficiency; $F_{28d}$ is the 28-day compressive strength of the shotcrete mortar. The specific greenhouse gas and acidifying gas emissions and potential factors for each material are shown in Table 4.

## 4. Conclusions

(1) Coal gangue sand, characterized by a porous structure, demonstrated significant effects on shotcrete mortar performance. At a 0.55 water–cement ratio with 100% replacement, the mortar exhibited 30% increased porosity, 40% decreased compressive strength, and 57% shortened setting time. Despite these changes, the mortar surface maintained integrity after 28-day water immersion, indicating basic durability.
(2) Optimal performance was achieved at 0.4 water–cement ratio with 50% coal gangue sand replacement, meeting M20 grade requirements with a setting time under 12 min and 28-day compressive strength exceeding 23 MPa. Notably, this mix design reduced environmental impact by 70% compared to conventional natural sand mortar at equivalent strength levels.
(3) Microstructural analysis revealed that coal gangue sand’s water absorption provided beneficial internal curing effects during cement hydration. Although pore size and volume increased by 20% and 30%, respectively, the improved interface transition zone and progressive cement hydration effectively compensated for the initial strength reduction, ensuring stable long-term performance.

## Figures and Tables

**Table 1:** Chemical composition of cement, coal gangue, and J85-type accelerator.

Chemical Composition	Na_2_O	MgO	Al_2_O_3_	SiO_2_	P_2_O_5_	SO_3_	K_2_O	CaO	TiO_2_	Fe_2_O_3_	Others
Natural sand	0.2	0.6	7.5	86.7	-	0.3	1.7	0.4	0.3	1.8	0.5
Coal gangue sand	1.3	0.8	24.3	63.0	0.2	0.3	3.8	1.2	0.9	4.1	0.2
J85-type accelerator	10.7	1.3	36.9	34.4	0.2	1.3	1.9	6.4	1.1	4.8	1.0

**Table 2:** Experimental mix proportions.

NO.	Cement (g)	Water (g)	Natural Sand (g)	Coal Gangue Sand (g)	Accelerating Agent (g)	Water Reducer (g)
GM0-0.55	900	495	2700	0	47.7	6
GM25-0.55	900	495	2025	675	47.7	8
GM50-0.55	900	495	1350	1350	47.7	10
GM75-0.55	900	495	675	2025	47.7	12
GM100-0.55	900	495	0	2700	47.7	15
GM0-0.4	900	360	2700	0	47.7	30
GM25-0.4	900	360	2025	675	47.7	32
GM50-0.4	900	360	1350	1350	47.7	35
GM75-0.4	900	360	675	2025	47.7	45
GM100-0.4	900	360	0	2700	47.7	60

**Table 3:** Life cycle inventory for cement, natural sand, and coal gangue sand.

Classification	Materials	Cement (kg/kg)	Natural Sand (kg/kg)	Coal Gangue Sand (kg/kg)	GWP/AP
Greenhouse gases	CO_2_	8.85 × 10^−1^	2.34 × 10^−3^	1.31 × 10^−2^	1
	CH_4_	5.80 × 10^−4^	3.70 × 10^−6^	2.34 × 10^−5^	27
	N_2_O	2.22 × 10^−5^	3.81 × 10^−8^	5.06 × 10^−5^	298
Acidified gas	SO_2_	1.05 × 10^−3^	9.49 × 10^−6^	6.55 × 10^−5^	1
	NO_x_	1.79 × 10^−3^	1.52 × 10^−6^	5.06 × 10^−5^	0.7
	NH_3_	1.02 × 10^−8^	7.24 × 10^−9^	5.37 × 10^−7^	1.88

**Table 4:** Global warming potential, acidification potential, and corresponding weight coefficients of cement, natural sand, and coal gangue sand based on calculations.

Classification	Cement (kg/kg)	Natural Sand (kg/kg)	Coal Gangue Sand (kg/kg)	Weight Coefficient (RMB/kg-CO_2eq_)
GWP	9.07 × 10^−1^	2.45 × 10^−3^	2.88 × 10^−2^	5.53 × 10^−2^
AP	2.30 × 10^−3^	1.06 × 10^−5^	1.02 × 10^−4^	6.57 × 10^−1^

**Table 5:** Effects of different coal gangue sand substitution rates on environmental impact and environmental strength-specific efficiency based on calculations.

NO.	${WTP}_{total}$	$k_{E-F}$
GM0-0.4	1.30 × 10^−2^	4.52 × 10^−4^
GM25-0.4	1.33 × 10^−2^	5.33 × 10^−4^
GM50-0.4	1.36 × 10^−2^	5.53 × 10^−4^
GM75-0.4	1.39 × 10^−2^	6.91 × 10^−4^
GM100-0.4	1.42 × 10^−2^	7.70 × 10^−4^

## Data Availability

The original contributions presented in this study are included in the article. Further inquiries can be directed to the corresponding author.
